# Radiolabeled Dendrimers for Nuclear Medicine Applications

**DOI:** 10.3390/molecules22091350

**Published:** 2017-08-25

**Authors:** Lingzhou Zhao, Meilin Zhu, Yujie Li, Yan Xing, Jinhua Zhao

**Affiliations:** 1 Department of Nuclear Medicine, Shanghai General Hospital, Shanghai Jiao Tong University School of Medicine, Shanghai 200080, China; zlz-330@163.com (L.Z.); 18701866757@163.com (Y.L.); xy.1@163.com (Y.X.); 2 Basic Medical College, Ningxia Medical University, Yinchuan 750004, Ningxia, China; jay70281@163.com

**Keywords:** dendrimers, nuclear medicine, PET, SPECT, radionuclide therapy, radiolabeling

## Abstract

Recent advances in nuclear medicine have explored nanoscale carriers for targeted delivery of various radionuclides in specific manners to improve the effect of diagnosis and therapy of diseases. Due to the unique molecular architecture allowing facile attachment of targeting ligands and radionuclides, dendrimers provide versatile platforms in this filed to build abundant multifunctional radiolabeled nanoparticles for nuclear medicine applications. This review gives special focus to recent advances in dendrimer-based nuclear medicine agents for the imaging and treatment of cancer, cardiovascular and other diseases. Radiolabeling strategies for different radionuclides and several challenges involved in clinical translation of radiolabeled dendrimers are extensively discussed.

## 1. Introduction

Nuclear medicine, the integration of physics [1], chemistry [2], engineering [3] and medicine [4], is regarded as one of the most powerful techniques for the diagnosis and therapy of diseases [4,5]. The origin of nuclear medicine can be traced back to the discovery of radioactivity by Henri Becquerel in 1896, however, it was only after several decades when the idea of using radionuclides as a medical tool was generated from George de Hevesy who performed the first radiotracer studies in animals to investigate dynamic processes in the body. As nuclear medicine continuously develops, the interest of researchers towards applying radionuclides in medical practices has been extensively risen [6,7,8,9]. Depending on the type of elements emitting from radionuclides, positron and gamma (γ) ray are applied for positron emission tomography (PET) and single photon emission computed tomography (SPECT) in nuclear medicine imaging, respectively, while alpha (α) and beta (β) particles can be used for nuclear medicine therapy [10,11,12]. Generally, these radionuclides need to be labeled with pharmaceutical molecules to form various radiopharmaceuticals [13,14]. In nuclear medicine therapy, therapeutic radiopharmaceuticals emit ionizing radiation rather than from an external radiation source, which transmits only a short distance while minimizing side effects and damages to noninvolved organs or nearby structures. Likewise, in nuclear medicine imaging, external detectors capture the signals emitting from diagnostic radiopharmaceuticals within the body to form images, which is unlike X-ray computed tomography (CT) recording radiation generated by external sources. In addition, nuclear medicine imaging is known as functional imaging but suffers from the intrinsic weakness of relatively poor spatial resolution [15,16]. With the advent of fusion imaging technique in nuclear medicine, this shortage has been compensated by other imaging modalities with high anatomical resolution, such as CT and magnetic resonance (MR) imaging [17,18,19]. The later formed hybrid imaging techniques, such as SPECT/CT, PET/CT and PET/MR imaging, vigorously promote the development of nuclear medicine for wider clinical applications [20,21,22]. Hundreds of different radiopharmaceuticals have been synthesized and tested in the last decade, however, few of them have been approved for clinical purposes, especially multifunctional nuclear medicine agents for fusion imaging techniques, and the efficacy and safety of radionuclide therapy have scarcely been further improved [12,23]. Therefore, it is essential to explore novel radiopharmaceuticals for this powerful technique.

Nanotechnology holds great promise to revolutionize the field of medicine and has brought challenging innovations in diagnosis and treatment of diseases, in particular building contrast agents for various imaging modalities and delivering biologically active substances to specific tissues or organs [24,25,26]. Recent advances in nanomedicine have shown that multiple types of nanoparticles (NPs) can be labeled with radionuclides for nuclear medicine imaging and therapy, such as liposomes, micelles, polymers, metal oxide NPs and dendrimers [27,28,29,30,31]. These NPs display improved diagnostic and therapeutic effects, lower toxicity and controllable biodistribution, compared with conventional small molecule radiopharmaceuticals. Among these developed NPs, dendrimers have been praised as an ideal candidate and attracted a great deal of attention due to the highly branched interior, well-defined architecture and abundant surface functional groups [31,32,33,34]. These unique structural features enable dendrimers not only to be efficiently labeled with various radionuclides, but also to conveniently construct multifunctional nanomaterials for achieving different nuclear medicine applications, such as dual or multimodality imaging and theranostics [35,36,37]. Meanwhile, the generation-dependent physical size and structure of dendrimers are frequently utilized to tune their excretion behavior and circulation time in vivo, as well as to acquire a suitable visualization of passive targeting behavior through enhanced permeability and retention (EPR) effect in specific areas, like tumors [38,39]. Furthermore, dendrimers are able to be functionalized with multiple targeting ligands to have higher probability to bind specific receptors overexpressed in tumor cells [40,41,42], and through appropriate surface modification, these ligand-modified dendrimers can acquire favorable water solubility and biocompatibility [43,44], which probably broadens the application of dendrimer-based radiopharmaceuticals in clinical use.

Although dendrimer-based NPs have been developed and gained encouraging results in many aspects, several key issues have to be considered before the construction of radiolabeled dendrimers. For instance, whether the purpose of radiolabeled dendrimers is imaging or therapy, considering the crucial role of physical half-life, the first step is selecting appropriate radionuclides. In order to achieve expected objectives, it is indispensable that the half-lives of selected radionuclides should be in harmony with the pharmacokinetic profiles of dendrimers. Commonly, the used radionuclides can be labeled through several strategies, and which radiolabeling method is more efficient depends on the composition and structure of dendrimers. This review focuses on the recent advances in dendrimer-based NPs for nuclear medicine imaging and therapy of cancer, cardiovascular and other diseases. In particular, radiolabeling strategies for different radionuclides are described in detail. Several challenges involved in clinical translation of radiolabeled dendrimers are also discussed.

## 2. Radiolabeled Dendrimers

Based on their medical applications, radionuclides can be divided into diagnostics and therapeutics. Diagnostic radionuclides contain γ-emitting isotopes in the energy range of approximately 75 to 360 keV for SPECT imaging and positron-emitting isotopes generating two 511 keV photons by annihilation for PET imaging. The high sensitivity of SPECT and PET imaging allows the dosage of radiopharmaceuticals at a range of 10^−6^ to 10^−8^ M [32]. Such low concentrations display no pharmacological effect, but can provide non-invasive techniques for in vivo real-time visualization, characterization and measurement of biological processes at the molecular and cellular levels [45,46,47], which allows clinical applications in disease diagnosis, prognosis evaluation and therapy monitoring [6,48]. Therapeutic radionuclides are almost all β and α-emitters, such as ^89^Sr, ^90^Y, ^131^I, ^153^Sm, ^177^Lu, ^188^Re, ^211^At and ^213^Bi [49]. Most of them have been currently used for the treatment of malignancies [50,51]. Ideally, they should be delivered to specific diseased sites and localize there with sufficient therapeutic doses of ionizing radiation, while clears rapidly from the blood stream and other normal organs or tissues to minimize radiation damage.

Due to the unique structural features, dendrimers can be efficiently labeled with various radionuclides in theory. Considering the physical half-life and radiolabeling strategies, dendrimers are mainly modified with bifunctional chelators (BFCs) on the surface and then labeled with radiometals via coordination chemistry [52,53,54,55]. ^99m^Tc and ^111^In are the typical SPECT isotopes in the construction of radiolabeled dendrimers, ^68^Ga and ^64^Cu are the most researched radionuclides for PET application, while ^177^Lu is regarded as a priority in the development of therapeutic dendrimers for radionuclide therapy. On the other hand, owing to well-established coordination chemistry, a series of BFCs have been designed and synthesized, which boosts the development of radiometals labeled dendrimers [56]. 1,4,7,10-Tetraazacyclododecane-1,4,7,10-tetraacetic acid (DOTA) is one of common chelators connected with dendrimers to load non-radioactive ^67^Gd(III) for MR imaging [57,58]. Moreover, diethylenetriaminepentaacetic acid (DTPA), 1,4,7-triazacyclononane-1,4,7-triacetic acid (NOTA) and 1,4,8,11-tetraazacyclotetradecane-*N*,*N*′,*N*′′,*N*′′′-tetraacetic acid (TETA) are additional candidates [59,60,61]. Apart from these radiometals, some radiohalogens such as ^76^Br, ^125^I and ^131^I can be conveniently labeled, for instance, via the chloramine T method by introduction of tyrosine into dendrimers [62]. ^18^F is the most important PET isotope in clinical use, however, radiolabeling of dendrimers with ^18^F is still complicated due to harsh reaction conditions, multistep protocols and low radiochemical yields in the traditional methods [63,64]. Therefore, novel radiolabeling strategies need to be developed for ^18^F-labeled dendrimers.

## 3. Radiolabeled Dendrimers for SPECT Imaging

**Figure 1:**
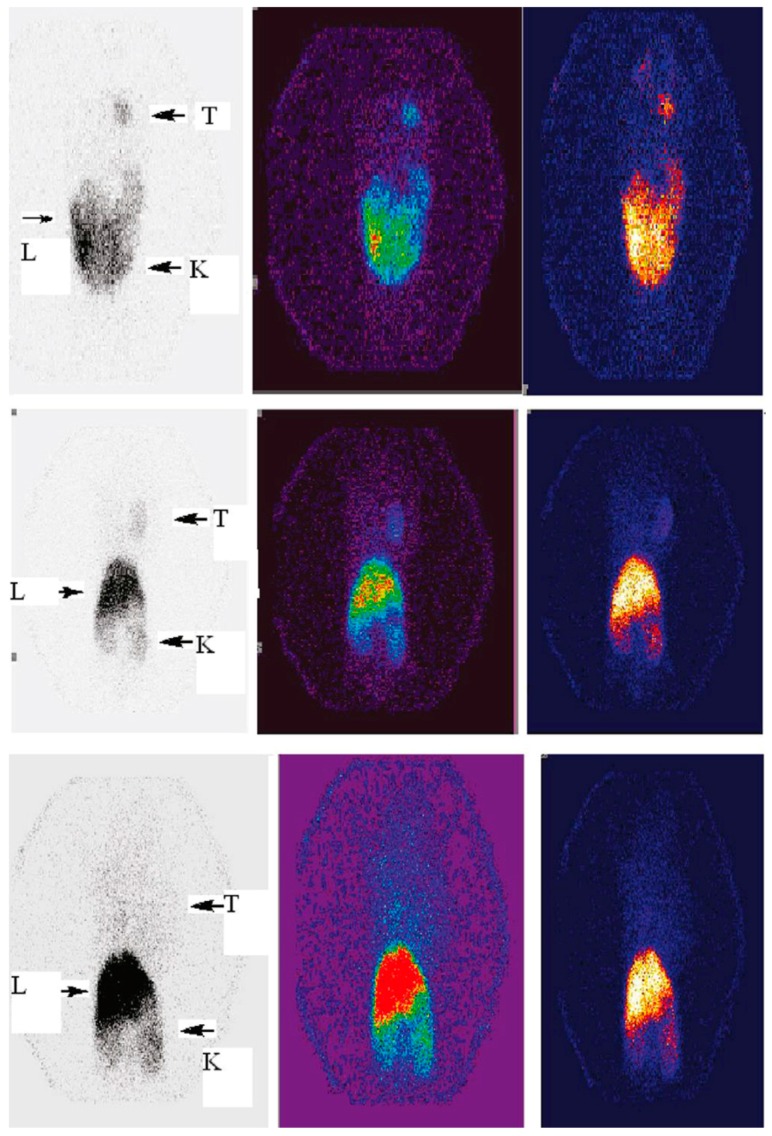
Micro-SPECT images of KB-bearing nude mice at 4 h: T, tumor; L, lungs; K, kidney ((**upper**) ^99m^Tc-G5-Ac-pegFA-DTPA; (**middle**) ^99m^Tc-G5-Ac-FA-DTPA; (**lower**) ^99m^Tc-G5-Ac-DTPA) (adapted from [66], Journal of Medicinal Chemistry, 2010).

^99m^Tc is by far the most commonly used radionuclide in SPECT imaging. This is due to its convenient acquisition from commercial ^99^Mo/^99m^Tc generators, latent chemical properties for radiolabeling and attractive physical properties including appropriate half-life (6.02 h) and energy γ-ray (140 keV), which is beneficial for both effective imaging and radiation safety [20]. The dendrimers conjugated with DTPA can be readily labeled with ^99m^Tc. For instance, Zhang et al. reported the synthesis and SPECT imaging of ^99m^Tc-labeled generation 5 (G5) polyamidoamine (PAMAM) dendrimers in folic acid (FA) receptor overexpressing tumor cells [65]. DTPA could be used as a chelator for ^99m^Tc with high radiochemical yield and stability. Preferential uptake of ^99m^Tc-labeled dendrimers in KB tumors were confirmed by biodistribution and micro-SPECT imaging studies. In their following studies, they demonstrated that PEGylated FA was able to further enhance the uptake of dendrimers in tumors compared to that of direct FA conjugation via EDC chemistry (Figure 1) [66], and the accumulation in kidneys could be observably decreased through employing avidin instead of FA but showed very high uptake in liver and spleen [67]. In addition to DTPA, hydrazinonicotinic acid (HYNIC) is another very efficient BFC for ^99m^Tc labeling. Recently, Song et al. showed ^99m^Tc radiolabeling of FA and HYNIC modified G3 PAMAM dendrimers [68]. The SPECT imaging displayed high accumulation of synthesized dendrimers in tumor and kidneys with low non-specific uptake in liver and lung. These satisfactory results will further promote the advancement of ^99m^Tc labeled multifunctional dendrimers.

**Figure 2:**
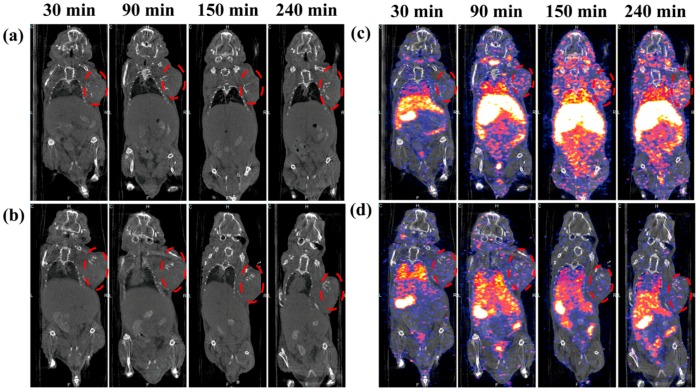
In vivo CT (**a**,**b**) and SPECT (**c**,**d**) images of tumors after intravenous injection of the {(Au°)_6_-G2-DTPA(^99m^Tc)-PEG-FA} (**a**,**c**) or {(Au°)_6_-G2-DTPA(^99m^Tc)-*m*PEG} (**b**,**d**) DENPs ([99mTc] = 740 MBq·mL^−^^1^, Au = 0.08 M, in 100 μL PBS) at different time points post-injection. The dashed red circles indicate the tumor sites (adapted from [73], ACS Applied Materials & Interfaces, 2016).

Dendrimer-based contrast agents possess great advantages in different imaging applications, including overcoming the drawbacks caused by small molecular iodinated or Gd(III)-based contrast agents and enhancing the fluorescence quantum yield for optical imaging [69,70,71]. The convenience of ^99m^Tc radiolabeling in dendrimers has enabled the development of various dual model imaging applications, such as SPECT/CT, SPECT/MR and SPECT/optical imaging. In an earlier study, Criscione et al. conjugated triiodinated moieties and ^99m^Tc on the surface of G4 PAMAM dendrimers for SPECT/CT application [72]. They found that the iodinated dendritic NPs displayed good X-ray attenuation properties, long enough intravascular residence time, and favorable contrast-to-noise ratio for serial intravascular and blood pool imaging. Recently, Shi and coworkers reported ^99m^Tc-labeled G2 PAMAM dendrimer-entrapped gold nanoparticles (Au DENPs) for tumor-targeted SPECT/CT imaging [73]. Biodistribution and SPECT/CT imaging studies demonstrated that the formed multifunctional Au DENPs had a great potential to be utilized as an effective and economic nanoplatform for dual-mode imaging of FAR-overexpressing tumors (Figure 2). In another investigation from the same group, Luo et al. developed a facile approach to prepare manganese (Mn) and ^99m^Tc-coloaded dendrimeric nanoprobes for tumor-targeted SPECT/MR imaging. G5 PAMAM dendrimers were used as a platform to link FA and DOTA which could complex with Mn(II) and ^99m^Tc simultaneously. Both SPECT and MR imaging showed that the dendrimer-FA conjugates were able to rapidly accumulate in tumors and achieve its peak value within 2 h, suggesting great potential of specific SPECT/MR imaging of cancer cells.

**Figure 3:**
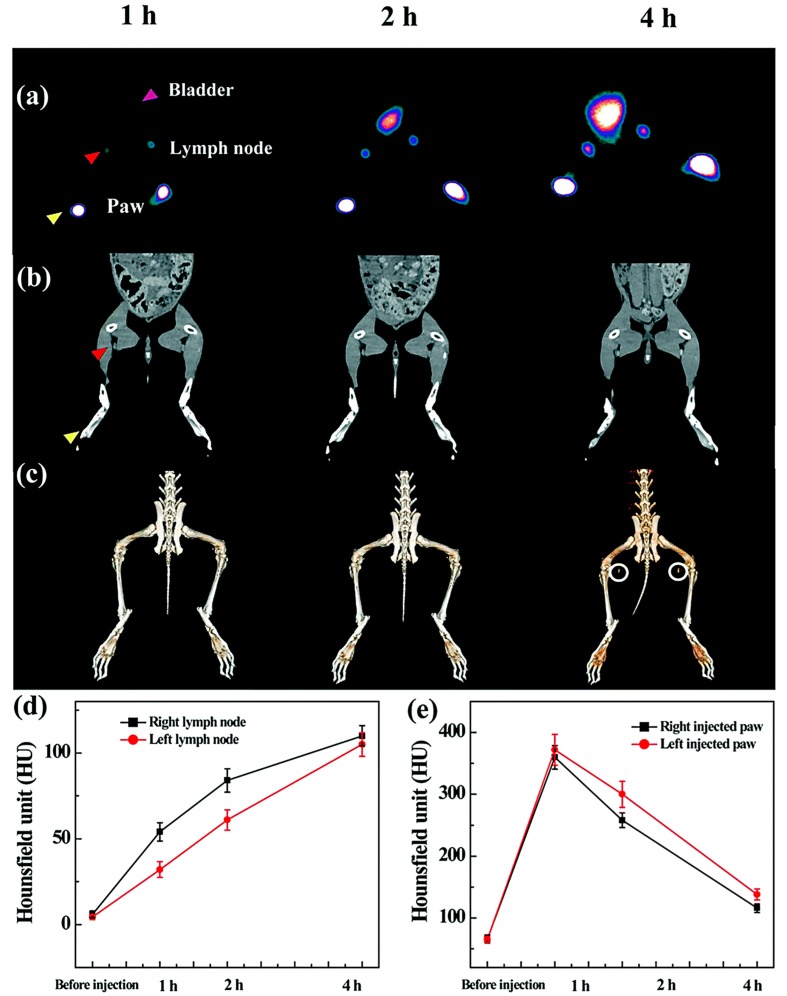
SPECT (**a**) and CT (**b**) imaging of a rabbit after hock injection of the ^99m^Tc-Au-Ac DENPs (left) and the ^99m^Tc-Au-Gly DENPs (right) at different time points and the corresponding 3D renderings of in vivo CT images (**c**); (**d**,**e**) Show the CT value of the lymph node and the injection paw before and at different time points post intravenous injection of the corresponding particles (adapted from [75], Journal of Materials Chemistry B, 2017).

The lymphatic system, especially the sentinel lymph node (SLN), plays a key role in cancer metastasis. Hence, noninvasive imaging of SNL using radiolabeled dendrimer-based NPs has attracted a great deal of attention in the field of cancer diagnosis and therapy. Tsuchimochi et al. developed G3 PAMAM dendrimer-coated silica NPs loaded with ^99m^Tc and indocyanine green (ICG) for SPECT/NIR imaging of SNL [74]. The formed dendrimers were injected into the tongue of rats, and then these NPs were able to clearly depict sentinel lymph nodes in real time via dual-modal SPECT/NIR imaging. Recently, Wen et al. reported ^99m^Tc-labeled dendrimer-entrapped gold NPs (Au DENPs) with different surface groups (acetyl or hydroxyl) for SPECT/CT imaging of SLN [75]. After respectively subcutaneous injection of ^99m^Tc-labeled acetyl or hydroxyl Au DENPs into the left and right paws of a rabbit, their accumulations in the popliteal lymph nodes could be clearly observed (Figure 3). Interestingly, during the period investigated, acetyl Au DENPs displayed steadily increased signals in lymph node, whereas the radioactivity of hydroxyl Au DENPs in SLN region gradually declined after 1 h post-injection. Biodistribution studies verified that surface groups had significant impact on their behaviors in vivo. Within 1 h after injection, acetyl Au DENPs were mainly accumulated in lung, liver and spleen, while hydroxyl Au DENPs could be found in the blood, heart and kidney, which allowed for preferential SPECT/CT imaging of different organs.

**Figure 4:**
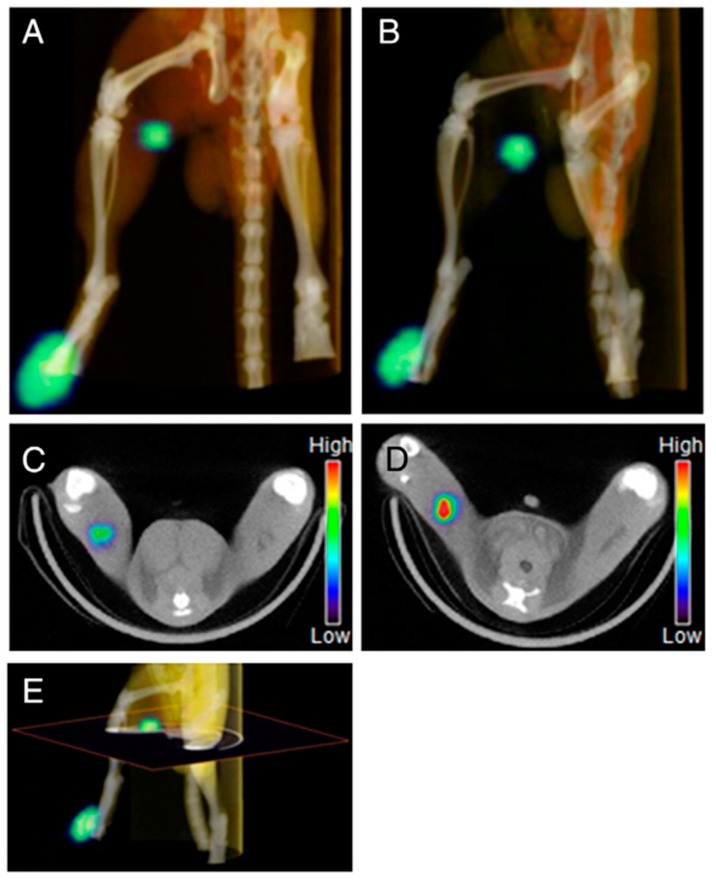
SPECT/CT images (**A**–**E**) after the injection of ^111^In-DTPA-G4/PEI (**A**,**C**) or ^111^In-DTPA-G4/PEI/γ-PGA (**B**,**D**) into footpads of SD rats (DTPA-G4: 10 μg/mL, 1.0–1.7 MBq/ 200 μL in 5% glucose/rat). Panels (**C**,**D**) are 2D transaxial images including lymph nodes constructed from 3D images (**A**,**B**) as shown in (**E**). ^111^In-DTPA-G4/PEI/γ-PGA (**B**,**D**) clearly visualized the popliteal lymph nodes (sentinel LNs in this model) compared to ^111^In-DTPA-G4/PEI (**A**,**C**) (adapted from [82], Journal of Controlled Release, 2014).

^111^In is another attractive radionuclide in SPECT applications, which can be efficiently produced by cyclotron [76]. ^111^In emits 173 and 247 keV γ rays with a relatively long half-life (2.8 days). Similar to ^99m^Tc, ^111^In can be effectively chelated by DTPA ligands. In earlier studies, Merkel et al. reported a family of triazine dendrimers as nonviral gene delivery systems with high transfection efficacy [77,78]. These flexible triazine dendrimer-based siRNA complexes were then synthesized for gene delivery systems and labeled with ^111^In via DTPA for SPECT imaging to identify efficient siRNA delivery in vivo [79]. Likewise, Chan et al. showed that G4 PAMAM dendrimers conjugated with DTPA and trastuzumab permitted high specific radioactivity for ^111^In labeling, and exhibited increased cytotoxic potency for breast cancer cells with high or low HER2 expression [80]. To monitor the in vivo behaviors of dendrimer-based drug delivery systems, Kojima et al. synthesized ^111^In-labeled DTPA-conjugated polymers using G4 acetylated PAMAM dendrimer (Ac-den) and collagen peptide-conjugated dendrimer (CP-den), and investigated their biodistribution in tumor-bearing mice [81]. These ^111^In-DTPA-bearing dendrimers were accumulated in liver and kidneys following intravenous administration, but largely retained at the injection site for at least 1 day through subcutaneous injection. Compared with Ac-den, CP-DTPA displayed longer retention time due to its higher molecular weight. These results indicated that the subcutaneously injected dendrimers might be used as drug depots around the injection site. Similarly, Sano et al. used G4 PAMAM as a template to prepare ^111^In-labeled dendrimers for SPECT imaging of SLN [82]. It seemed that γ-polyglutamic acid (γ-PGA) could improve the uptake of synthesized nanoprobes in macrophage cells in vitro due to the mechanisms of phagocytosis and γ-PGA specific pathway. Micro-SPECT imaging studies further confirmed that after intradermal administration into footpads of rats, γ-PGA modified dendrimers had a relative fast clearance from the injection site and significantly higher radioactive uptake in the first draining popliteal LN comparable to ^111^In-labeled dendrimers without γ-PGA modification (Figure 4). Subsequently, Niki et al. systematically studied ^111^In-labeled different generation (G2, G4, G6 and G8) dendrimers with various terminal groups (amino, carboxyl and acetyl) to determine the optimal structure for SLN imaging [83]. The results showed that high generation (greater than G4) PAMAM dendrimers with carboxyl-termini were able to significantly accumulate at the SLN for SPECT imaging, which might have an important effect on the development of dendrimer-based SLN imaging agents and SLN-targeted drug carriers.

^125^I is a radioisotope of iodine with low energy γ-ray (35 keV) which is poorly suited for clinical SPECT imaging, but very useful for radioimmunoassay test, implantation therapy and preclinical study due to its long half-life (60.1 days) [84,85]. Many earlier researches on ^125^I-labeled dendrimers for SPECT or biodistribution studies have been reviewed elsewhere [32]. In a recent work, Xiao et al. reported a multifunctional telodendrimer-based micelle system for delivery of chemotherapy agents, and the biodistribution and pharmacokinetic data were obtained by SPECT/CT imaging of ^125^I-labeled telodendrimer in a ovarian cancer mouse model [86]. In another study, Lee et al. prepared a G3 triazine dendrimer with 8 PEG chains and 16 paclitaxel groups for drug delivery [87]. The paclitaxel bearing dendrimers could be simply labeled with ^125^I through a Bolton-Hunter moiety. Biodistribution and SPECT/CT imaging of ^125^I labeled complexes suggested significant persistence in the vasculature with slow clearance and high tumor uptake while showing low levels of radiolabeled dendrimer in lung, liver and spleen.

## 4. Radiolabeled Dendrimers for PET Imaging

### 4.1. Cancer Imaging

**Figure 5:**
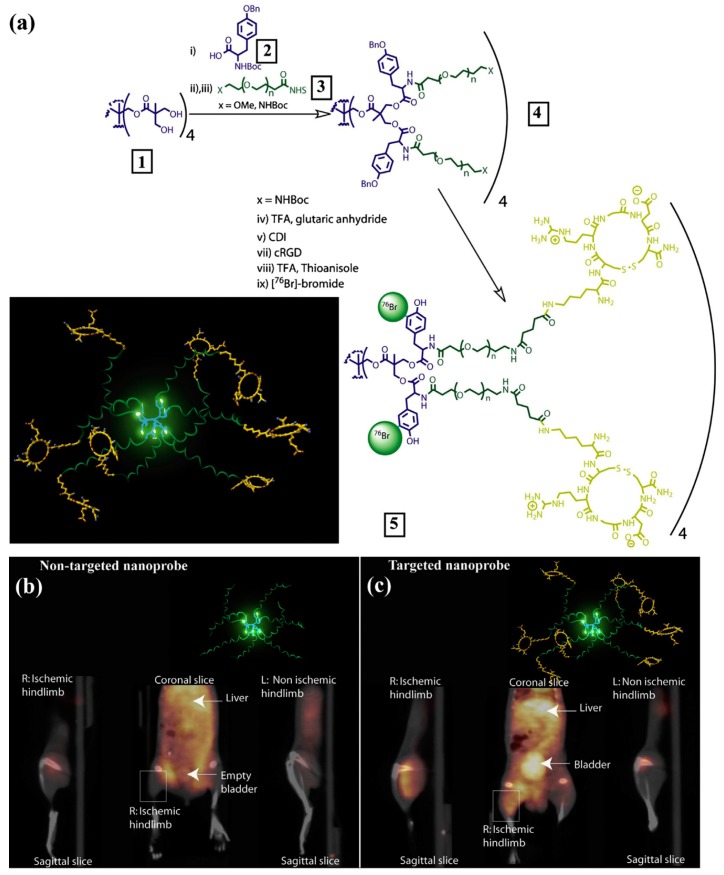
(**a**) Preparation of PET nanoprobes targeted at α_v_β_3_ integrin; (**b**) Noninvasive PET/CT images of angiogenesis induced by hindlimb ischemia in amurine model for nontargeted dendritic nanoprobes (shown bottom center); (**c**) Noninvasive PET/CT images of angiogenesis induced by hindlimb ischemiain a murine model for α_v_β_3_-targeted dendritic nanoprobes, which showed higher uptake in ischemic hindlimb (left side of image) as compared with control hindlimb (right side of image) (adapted from [31], Progress in Polymer Science, 2015).

Some common clinically used radionuclides for PET imaging are ^11^C, ^13^N, ^15^O and ^18^F [88]. Because of the very short half-lives of ^11^C, ^13^N and ^15^O (2 to 20 min), they are mainly used for measurements within an initial time frame and only a very few labeled NPs have been reported [89,90]. Although ^18^F is regarded as an ideal positron emitter for PET imaging and abundant ^18^F labeled agents have been developed for different clinical applications in the past decades, the addition of ^18^F to macromolecules is still challenging and a number of alternative labeling strategies have been developing for the efficient synthesis of ^18^F-labeled NPs [63,64]. Trembleau et al. first showed that dendrimers could be labeled with ^18^F-fluorinatable groups at room temperature [91]. The dendrimers were designed to possess a disulfide linkage that subsequently generated two dendrons with thiol groups for conjugation of biotin. Trifluoroboroaryl moieties were connected with the terminal NH_2_ groups of dendrimers to enable ^18^F radiolabeling at room temperature in aqueous solvent. These biotin functionalized dendrimers showed high specificities to HER-2 expressing cells in vitro. In comparison to ^18^F, ^76^Br (16.2 h) has a relatively long half-life and can be labeled to macromolecules in a simple way. Several researchers have reported ^76^Br-labeled antibody with high yield using Chloramine-T method [92,93]. With the same method, Almutairi et al. built ^76^Br-labeled biodegradable dendrimers for PET imaging of angiogenesis (Figure 5) [94]. The dendrimers used pentaerythritol as a core to modify with tyrosine groups for the radiolabeling of ^77^Br. Heterobifunctional polyethylene oxide (PEO) chains were conjugated to the periphery of dendrimers and formed protective shells to prevent in vivo dehalogenation. Lysine modified RGD peptides were installed at the ends of PEO chains to increase the specificity of dendrimers. Remarkably, the pharmacokinetic profiles were able to be modulated via appropriate level of dendritic branching and length of PEO chains. Compared with nontargeted nanoprobes, the targeted nanoprobes exhibited 6-fold increase in α_v_β_3_ receptor-mediated endocytosis and a 50-fold enhancement in binding affinity over the mono-RGD peptide. Selective accumulation of ^76^Br-labeled dendritic nanoprobes was significantly observed in a murine hindlimb ischemia model and the feasibility for PET imaging of angiogenesis was also verified in vivo.

**Figure 6:**
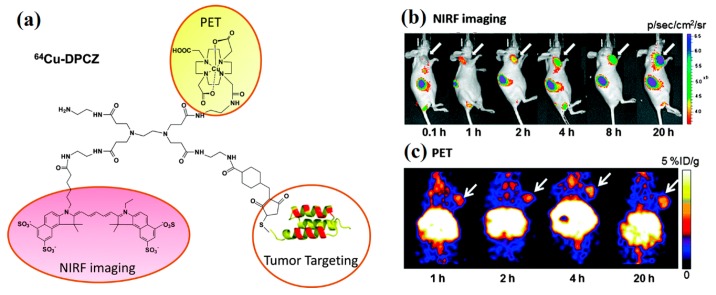
(**a**) Schematic structure of ^64^Cu-DPCZ which is constituted by four components, PAMAM G0 as a scaffold, Cy5.5 as an optical reporter, ^64^Cu-DOTA as a PET reporter and Affibody as a tumor targeting molecule; (**b**) In vivo NIRF imaging of SKOV3 tumor-bearing mice at 0.1, 1, 2, 4, 8, and 20 h after tail vein injection of ^64^Cu-DPCZ; (**c**) Decay-corrected coronal micro-PET images of mice bearing SKOV3 tumor at 1, 2, 4 and 20 h after tail vein injection of ^64^Cu-DPCZ. Arrows indicate the location of the tumors (*n* = 3) (adapted from [97], Chemical Communications, 2014).

^64^Cu and ^68^Ga are the most extensively researched and utilized radiometals in the construction of radiolabeled NPs for PET imaging because of handy radiolabeling methods and favorable decay half-lives [95,96]. ^64^Cu is generally produced by cyclotron accelerator and ^68^Ga can be acquired from a commercial ^68^Ge/^68^Ga generator. As the ^99m^Tc and ^111^In radiometals mentioned above, BFCs are required to attach ^64^Cu and ^68^Ga to NPs, including DOTA, NOTA and TETA. Wang et al. used PAMAM generation 0 (PAMAM G0) as a platform to assemble ^64^Cu and Cy5.5, and developed an anti-HER_2_ Affibody-based nanoprobe for dual-modality imaging of ovarian cancer (Figure 6) [97]. Both NIRF and PET imaging displayed high tumor accumulations in vivo at 1 h post injection, and thanks to the favorable pharmacokinetic properties, excellent tumor imaging effects could be observed within 20 h. Interestingly, tumor fluorescence signals gradually increased during the period investigated, whereas a radioactivity peak from PET were found at 4 h after injection. Biodistribution studies showed that the dendrimer-based nanoprobe primarily accumulated in liver and kidneys, indicating the excretion through both hepatobiliary and kidney systems. In another study, Li et al. developed smart and versatile telodendrimers consisting of various imaging and therapeutic functions containing NIRF, PET and MR imaging, photothermal therapy (PTT), photodynamic therapy (PDT) and imaging-guided drug delivery [98]. This “all-in-one” nano-platform was synthesized by the self-assembly of hybrid amphiphilic polymers which processed an intrinsic ability to chelate ^64^Cu for PET imaging and Gd(III) for MR imaging. In the presence of sodium dodecyl sulphate (SDS), the telodendrimers exhibited strong red-fluorescence emissions at 680 nm and possessed the ability of photodynamic transduction which could convert light in the form of fluorescence and singlet oxygen generation for NIRF imaging and PDT, but light to heat in phosphate-buffered saline (PBS) for PTT. Furthermore, chemotherapeutic drugs could be efficiently encapsulated inside telodendrimers as programmable releasing nanocarriers for drugs delivery, which had been have demonstrated in both ovarian cancer xenograft and murine transgenic breast cancer models in vivo.

**Figure 7:**
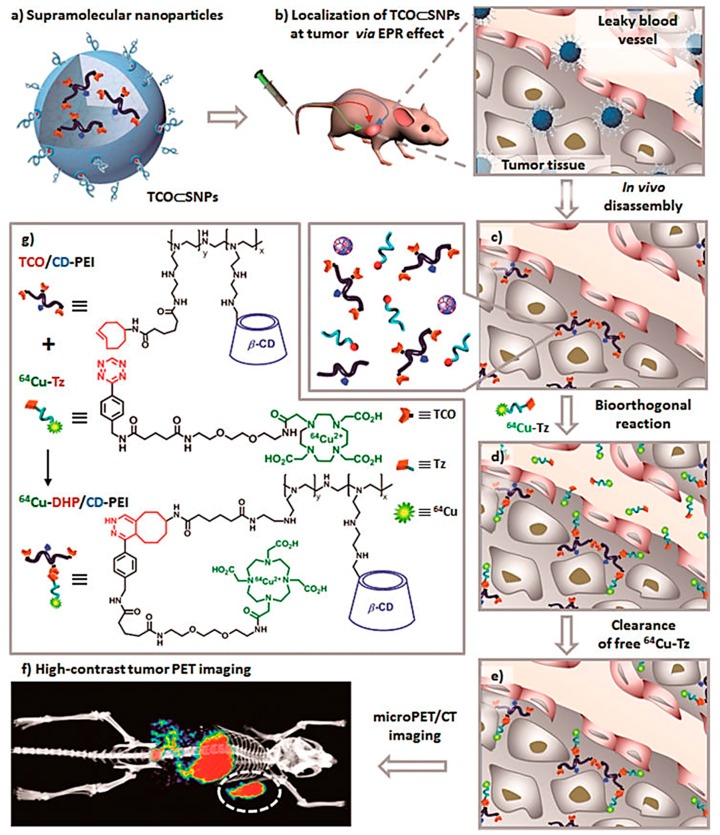
Schematic representation of a new approach for pretargeted PET imaging that leverages the utilities of supramolecular nanoparticles (SNPs) and bioorthogonal chemistry: (**a**) Supramolecular synthetic strategy is employed for preparing the tumor-targeting agent (TCO ⊂ SNPs); (**b**) after intravenous injection, the tumor EPR effect drives preferential accumulation of TCO ⊂ SNPs in tumor; (**c**) after TCO ⊂ SNPs have accumulated in tumor, TCO ⊂ SNPs disassemble to release a TCO-grafted molecular building block, TCO/CD-PEI; (**d**) a radiolabeled reporter (^64^Cu-Tz) is then injected for bioorthogonal reaction with tumor-retained TCO/CD-PEI; (**e**) the unreacted ^64^Cu-Tz was cleared quickly from the body; (**f**) the resulting dihydropyrazine (DHP) conjugation adduct (^64^Cu-DHP/CD-PEI) confines radioactivity in tumor, resulting in high-contrast tumor PET imaging. (**g**) Chemical structures of the bioorthogonal reactions between TCO/CD-PEI and ^64^Cu-Tz (adapted from [101], ACS Nano, 2016).

The application of PET nanoparticles is prone to be immersed in the contradiction between intrinsic pharmacokinetics (PKs) of NPs and limited half-lives of positron-emitting isotopes. For this problem, the pretargeted imaging strategy could probably be an effective solution and has been well established for several decades [99,100]. In a typical pretargeted PET imaging system, tumor-targeting agents firstly accumulate in tumors within a reasonable time frame, and then radiolabeled ligands irreversibly and selectively combine these tumor-targeting agents previously accumulated in tumors. Meanwhile, uncombined radioligands eliminate rapidly from the body to obtain optimal tumor PET imaging. In a recent study, Hou et al. reported ^64^Cu-labeled supramolecular NPs for pretargeted PET imaging (Figure 7) [101]. These supramolecular NPs contained a transcyclooctene (TCO) motif to label ^64^Cu via Diels-Alder reaction between TCO and tetrazine-DOTA-^64^Cu (^64^Cu-Tz). To avoid potential in vivo degradation, TCO groups were initially encapsulated into supramolecular NPs. When preferential accumulation in tumor sites occurred through EPR effect, the supramolecular NPs disassembled and released TCO to react with the subsequently injected ^64^Cu-Tz. The unreacted ^64^Cu-Tz were cleared quickly from the body, resulting in high-contrast tumor PET imaging. In this pretargeted imaging approach, approximately equivalent uptake in tumor and liver were observed, which superbly improved the imaging performance in contrast to traditional nanoparticle-based imaging platforms end up with low tumor uptake and high liver distribution.

### 4.2. Other Applications

Besides cancer imaging, PET imaging also has a wide application for cardiovascular and inflammatory diseases [102,103]. Especially in current clinical applications, some PET imaging agents have been regarded as gold standards for clinical research and objective assessment, such as ischemic disease and the extent of myocardial viability. Considering the unique advantages of dendrimers, many efforts have been made to develop sensitive and rapid methods for early detection in this filed. For instance, Seo et al. found that a cyclic 9-amino acid peptide (LyP-1) could bind to p32 protein and serve as a biomarker in the progression of atherosclerosis, but the binding affinity of LyP-1 was not strong enough in aorta [104,105]. To increase the binding avidity in atherosclerosis, they designed and synthesized a dendritic form of LyP-1 using lysine as the core. An analogue of TETA (6-BAT) was attached to the dendrimer as BFC for radiolabeling of ^64^Cu [106]. The ^64^Cu-labeled dendrimer with multiple LyP-1 ligands showed significantly enhanced accumulation in atherosclerotic plaque and higher aorta/blood ratio as compared with both the monomer and control peptide in vivo PET imaging. In another study, Pant et al. explored ^64^Cu-labeled dendritic polyglycerol sulfates (dPGS) as inflammation-specific PET imaging agents [107]. Notably, two novel types of copper(II)-chelating ligands were prepared via facile modification of 1,4-bis(2-pyridinylmethyl)-1,4,7-triazacyclononane (DMPTACN) with isothiocyanate or maleimide groups. They could directly couple with the amino or sulfhydryl groups of dPGS to form dPGS-DMPTACN complexes which were able to efficiently chelate ^64^Cu with high yield and excellent in vitro stability. However, PET imaging and biodistribution studies of the ^64^Cu-labeled dPGs were only performed in normal rats, and the potential of these inflammation-specific agents should be further investigated in inflammatory models.

**Figure 8:**
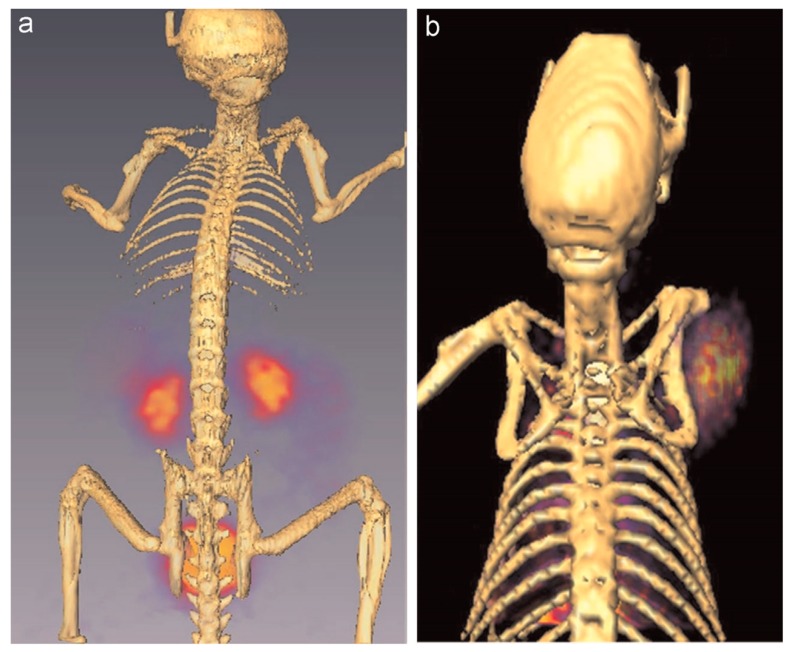
(**a**) Reconstructed PET/CT imaging in normal Balb/c mice with ^68^Ga-DOTA–PAMAM-D; (**b**) Reconstructed PET/CT imaging in EAT bearing Balb/c mice with ^68^Ga-DOTA–PAMAM-D. Reproduced with permission from (adapted from [110], Applied Radiation and Isotopes, 2015).

^68^Ga is a non-physiologic metallic positron emitter but has attracted great attention because of suitable half-life of 67.8 min, advantageous radiolabeling methods and low production cost [108]. Similar to ^64^Cu, ^68^Ga can also be chelated with DOTA and NOTA, but has better image quality than ^64^Cu in theory due to its higher positron decay proportion (89% vs. 17.8%). Tanaka et al. reported the first PET imaging of ^68^Ga-labeled dendrimer-type clusters containing 16 molecules of asparagine-linked oligosaccharide (*N*-glycan) to visualize their dynamics and biodistributions in normal mice. [109]. The hexadeca-clusters (16-mers) could be derived three structures due to different types of *N*-glycans which were bis-Neua(2-6)Gal-containing glycan (a), asialo glycan (b) and bis-Neua(2-3)Gal-glycan (c), respectively. PET imaging showed similar biodistributions in the initial stages but significantly different clearance properties among these 16-mers. 16-mer-a was slowly eliminated from kidney/urinary bladder and gallbladder (intestinal excretion pathway), while 16-mer-b and 16-mer-c were rapidly cleared through the kidney to the bladder. Because asialoglycoprotein receptors are highly expressed in liver, some accumulation of 16-mer-b was observed in this organ. These results suggested that the Neua(2-6)Gal linkage played an important role in the circulatory residence of *N*-glycans and markedly differentiated the clearance pathway from those of glycoclusters 16-mer-b and 16-mer-c, which proceed through a biofiltration pathway in kidneys. In addition, clusters consisting of 4 and 8 molecules of bis-Neua(2-6)Gal-containing glycan were also prepared. Due to their smaller molecular sizes, the formed 4-mer and 8-mer could be rapidly and almost completely cleared through kidney. Recently, Ghai et al. described the optimal radiolabeling method for ^68^Ga conjugated PAMAM G4 dendrimer-DOTA [110]. The best radiolabeling efficiency was achieved at pH 4.0, 30 min of incubation time and reaction temperature between 90 to 100 °C. PET imaging showed that this ^68^Ga-labeled dendrimers could be efficiently retained in tumor tissues through EPR effect and excreted primarily through kidneys (Figure 8).

Arginine-Glycine-Aspartic Acid (RGD) peptide is well known as a specific ligand with high affinity for α_v_β_3_ integrin which is frequently overexpressed on activated endothelial cells of growing vessels and several tumor cells such as melanoma, glioma, lung, ovarian and breast cancers. Noninvasion imaging of α_v_β_3_ integrin using radiolabeled cyclic RGD (cRGD) peptide has always been the focus in field of cancer and cardiovascular diseases [111,112,113,114,115]. However, the applicability of monomeric cRGD peptide is limited because of low tumor accumulation and rapid tumor washout. Compared with monomers, cRGD multimers exhibit enhanced binding affinity and selectivity in vivo, which further improves the uptake and retention characteristics in tumor region. Several groups have investigated radiolabeled cRGD multimers containing two or four cRGD moieties as agents for PET imaging applications. Tetrameric cRGD peptides showed higher affinity and specificity than dimers to tumor cells. Based on multivalency effect, the integrin-binding affinity was further improved in a cRGD octamer, resulting in higher initial uptake and longer tumor retention [115]. Subsequently, Wängler et al. applied PAMAM dendrimers as scaffold structures to manufacture various cRGD peptide multimers by click chemistry and first reported the hexadecimers [116]. These cRGD peptide multimers were further derivatized with PEGylated DOTA for ^68^Ga labeling. As expected, the binding affinities of the RGD multimers constantly amplified with increasing number of peptide moieties in vitro studies. Relative to the monomer, the affinities of hexadecimers to immobilized α_v_β_3_ integrin and U87MG cells were up to 131 and 124 times, respectively. In the following work, this synthesis approach using dendrimer as scaffold structures for radiolabeled bioactive multivalent molecules was further applied for other peptides [117,118]. For instance, PESIN peptide is regarded as a promising ligand to gastrin releasing peptide receptor (GRPR) which is overexpressed on various tumors. Lindner et al. synthesized ^68^Ga-labeled monomers and multimers (dimers, tetramers and octamers) of PESIN ligands on dendrimer scaffolds comprising PEG linkers of different lengths for PET imaging of GRPR overexpressing tumors. The highest binding affinities in vitro were found within each group (monomers to octamers) for the peptides modifying with the shortest PEG linker. Differently, the trend that binding affinities steadily increased with the number of peptide moieties did not occurred in the case of PESIN multimers. The dimers showed the optimized results, achieving a 2.5-fold avidity enhancement in vitro, and a twice higher tumor uptake in tumor-bearing mice compared to the respective monomers.

## 5. Radiolabeled Dendrimers for Radionuclide Therapy

The rapid development of nuclear medicine and dendrimer-based nanoparticles has offered opportunities for targeted radionuclide therapy [119,120]. In this area, a convenient way is to use therapeutic radionuclides instead of diagnostic radionuclides in existing nanoprobes. It is important to note that some therapeutic radionuclides also emit γ rays with suitable energy for SPECT imaging, providing a handy way to monitor the progress of treatments [121,122]. To date, a great variety of isotopes become available for radionuclide therapy, such as ^131^I, ^188^Re and ^177^Lu. Among them, ^131^I is one of the most common therapeutic radionuclides in clinical use, because of its relatively long half-life (8.01 days) and appropriate beta radiation energy (606 keV) for radiotherapy [123]. Moreover, ^131^I emits a γ-ray (364 keV) for SPECT imaging which renders its feasibility for theranostic applications. In a recent study, Shi group reported a series of multifunctional dendrimers labeled with ^131^I for targeted SPECT imaging and radiotherapy of different cancers [123,124,125]. In these studies, G5 amine-terminated PAMAM dendrimers were used as platforms to be sequentially conjugated with PEG, targeting agent biotoxins or FA, and 3-(4-hydroxyphenyl)propionic acid-OSu (HPAO). These were followed by acetylation of the remaining dendrimer terminal amines and radiolabeling with ^131^I directly through HPAO to form the targeted theranostic dendrimeric nanoplatforms. The formed ^131^I-labeled multifunctional dendrimers with good cytocompatibility and organ compatibility could be used as promising nanoplatforms for SPECT imaging and radiotherapy of different types of MMP2 or FAR-overexpressing cancers.

**Figure 9:**
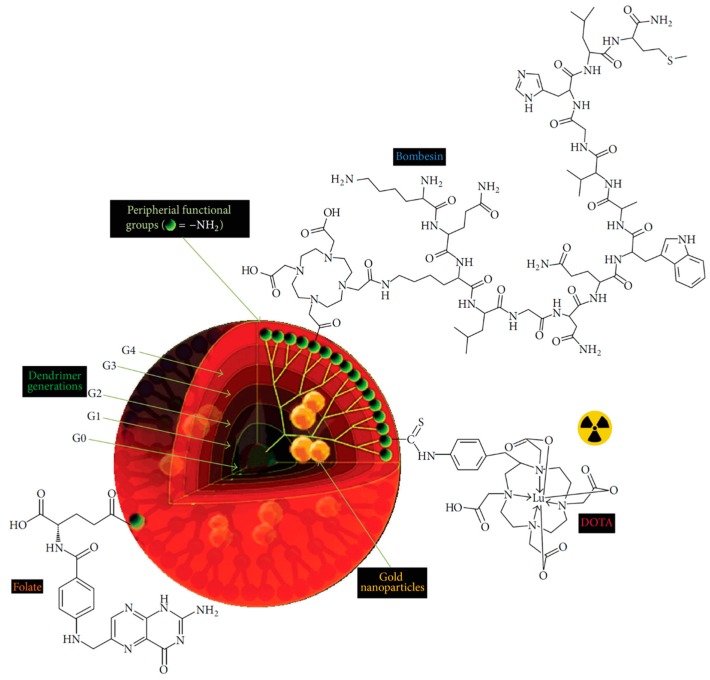
Overall scheme of ^177^Lu-DenAuNP-folate-bombesin (adapted from [134], Journal of Nanomaterials, 2016).

^188^Re is another commonly used therapeutic radionuclide in nuclear therapy [126]. ^188^Re has favorable physical properties, including its short half-life (16.9 h) and β photo emission of 2.12 MeV for distance of 12 mm in tissue and γ emission of 155 keV, which is very suitable for both effective therapy and imaging. Similar to ^99m^Tc, ^188^Re can also be readily derived as a column elute from ^188^W/^188^Re generator and effectively conjugate with DTPA [127,128]. Cui et al. reported ^188^Re radiolabeling of FA conjugated G5 PAMAM dendrimers [129]. The labelling yield was 67.1% and high in vitro stability. However, in vivo stability should be further improved and no therapeutic study could be found in this literature. In recent decades, ^177^Lu has been as a promising isotope used for diagnostic and therapy in basic research and clinical applications [130,131]. The half-life time of ^177^Lu is about 1 week (6.65 days) with β emitting (E_max_ = 497 keV) at a maximum tissue penetration of 2 mm for therapy and emits low-energy γ emitting for imaging. DOTA and its derivatives are regularly used as chelators for ^177^Lu radiolabeling. Recently, Laznickova et al. reported that a DOTA analog with one methylene pyridine-*N*-oxide pendant arm (DO3A-py^NO-C^) could serve as bifunctional chelators with stronger chelate ability than DTPA and EDTA [132]. In this study, ^177^Lu labeled G1 and G4 dendrimer conjugates could be prepared with a high specific activity and radiochemical purity. Several factors on radiolabeling efficacy were well investigated, including pH, reaction temperatures and chelator concentration. The optimum reaction conditions might be the molar ratio of DO3A-py^NO-C^ to ^177^Lu greater than about 8 × 10^6^, pH 5.6 and 40 °C. These radiolabeled dendrimers displayed excellent stability in vitro and different behaviors in vivo. The clearance of ^177^Lu labeled G1 dendrimers was rapid with no specific radioactivity uptake in organs and tissues in normal rats, while the elimination of G4 dendrimers was moderated with high and prolonged hepatic and renal accumulation. In a following study, Kovacs et al. studied the biodistribution of ^177^Lu-labeled G4 PAMAM dendrimers in tumor-bearing mice (Figure 9) [133]. As expected, high hepatic and renal accumulation were found, and the tumor uptake was triggered through the EPR effect. Interestingly, the elementary changes in tumor tissue was measured to employ as indicators of damage caused by ionizing radiation of ^177^Lu. In another study, Mendoza-Nava et al. synthesized ^177^Lu labeled G4 PAMAM dendrimer entrapped gold nanoparticles in the dendritic cavity for tumor imaging and radionuclide therapy [134]. Folate and bombesin were conjugated on the surface of dendrimers to target the folate and gastrin-releasing peptide receptors overexpressing breast cancer cells. The theranostic dendrimers had specific uptake in breast cancer cell and high retention in tumor sites in mice after intratumoral administration.

## 6. Conclusions and Outlooks

Nuclear medicine currently has been an essential tool in the diagnosis and treatment of various diseases, however, is vulnerable to be restricted by the insufficient radiopharmaceuticals. Thanks to the unique structural features and rapid development of dendrimers, abundant new radiopharmaceuticals have been explored. In this review, we have presented the typical examples of dendrimer-based nanoparticles for nuclear medicine applications including SPECT imaging, PET imaging and radionuclide therapy. Moreover, dendrimer-based nanoparticles can be as platforms to generate CT, MR and optical imaging agents or load chemotherapeutics for drug delivery, which enables the radiolabeled dendrimers for dual or multimodality imaging, theranostics and image-guided drug delivery. The multifunctional dendrimers have been used in many biological systems, such as blood pool, lymph nodes, major organs and cancer. Notably, these developed dendrimer-based imaging agents can be further modified with targeting ligands to improve specificity and decrease the non-specific accumulation.

Despite plenty of investigation on dendrimer-based nanoplatforms and encouraging outcomes, a great number of problems need to be explored in their clinical translation. The primary barrier is lack of admirable specificity in target tissues and excessive uptake by mononuclear phagocytic system, which leads to inevitable issues on the toxicity of dendrimer-based nanoparticles, particularly the macromolecular systems with slow elimination and latent damages from long-lived therapeutic radionuclides labeled dendrimers. One promising solution to increase targeting specificity is to modify highly specific ligands with dendrimer platforms, such as monoclonal antibody that is able to recognize specific receptors or antigens in vivo. On the other hand, the in vivo biodistribution behavior of dendrimers can be optimized via regulation of physical properties to prolong retention time in target location and increase the clearance from undesired tissues or organs. In addition, further types of dendrimer-based nanoparticles should be developed in order to satisfy different requirements. For instance, to expand the scope of nuclear medicine imaging, radionuclides can be modified on the surface of dendrimer-based iron oxide NPs for nuclear medicine imaging and T_2_-weighted MR imaging. Meanwhile, novel radiolabeling strategies with sufficient radiochemical yields and in vivo stabilities must be developed for these dendrimers. Particularly, several promising labeling strategies should be applied in the construction of ^18^F-labeled dendrimers. Taking click chemistry as an example, through the copper-catalyzed azide-alkyne cycloaddition reaction [135], ^18^F can be efficiently and mildly conjugated to azide-modified dendrimers. Lastly, for the capacity of dendrimers to load drugs, genes and therapeutic radionuclides, other types of dendrimer-based theranostic systems should be developed in order to expand the scope of nuclear medicine applications. In conclusion, with the development of nanotechnology, we expect all these challenges will be easier to meet and novel radiolabeled dendrimers will allow precise disease management.
